# Analgesic Effectiveness of Ultrasound-Guided PECS II Block in Implantable Venous Port Catheter Placement: A Retrospective Comparative Study

**DOI:** 10.3390/medicina62101877

**Published:** 2026-09-28

**Authors:** Ümit Karatepe, Ayse Azak Bozan, Mesut Oterkus, Cihan Doger

**Affiliations:** 1 Department of Anaesthesiology and Reanimation, Elazığ Fethi Sekin City Hospital, 23280 Elazig, Türkiye; ayseazak23@hotmail.com; 2 Department of Anesthesiology and Reanimation, Faculty of Medicine, Malatya Turgut Özal University, 44210 Malatya, Türkiye; mesut.oterkus@ozal.edu.tr; 3 Department of Anesthesiology and Reanimation Ankara, Ankara Bilkent City Hospital, 06800 Ankara, Türkiye; cihandoger@gmail.com

**Keywords:** PECS II block, venous port catheter, postoperative pain, ultrasound, regional anaesthesia

## Abstract

*Background and Objectives*: Total implantable venous access port (TIVAP) placement is a frequently performed but potentially painful procedure in oncology patients. This study aimed to compare the analgesic effectiveness, sedation requirements, and patient comfort associated with ultrasound-guided PECS II block versus conventional local infiltration anesthesia during TIVAP placement. *Materials and Methods*: Data from 118 patients who underwent TIVAP placement between 2021 and 2024 were retrospectively reviewed. Patients were divided according to the anesthesia method used: local infiltration (*n* = 64) and ultrasound-guided PECS II block (*n* = 54). The primary outcome was postoperative pain, assessed using the Visual Analog Scale (VAS), while secondary outcomes included requirements for additional local anesthetic, sedation and rescue analgesia, patient satisfaction, hemodynamic parameters, and procedure-related complications. *Results*: PECS II block was associated with lower VAS scores during the early postoperative period. Sedation was required in 11.1% of patients in the PECS II group and 56.3% in the infiltration group, while rescue analgesia was required in 11.1% and 82.8%, respectively (both *p* < 0.001). Patient satisfaction was higher in the PECS II group (*p* < 0.001). Any recorded procedure-related complication occurred in 7.4% of patients in the PECS II group and 20.3% in the infiltration group (Pearson chi-square *p* = 0.047); individual complication types were infrequent. *Conclusions*: In this retrospective cohort, PECS II block was associated with lower postoperative pain scores and reduced requirements for sedation and rescue analgesia compared with local infiltration anesthesia. These findings support PECS II block as a potential component of multimodal analgesia for TIVAP placement, while prospective randomized studies are needed for confirmation.

## 1. Introduction

Central venous catheterization is a frequently used procedure, particularly in patients receiving systemic chemotherapy, in cases requiring prolonged hospitalization, in situations where the peripheral venous system is unavailable, when palliative care is needed, and in patients for whom total parenteral nutrition is indicated. Upon being defined for intravenous chemotherapy in oncology patients in the 1980s, subcutaneous implantable port systems were introduced and began to be used [1].

The most important feature of port catheters is their low infection probability due to the absence of an external interfascial extension, together with increased patient comfort because daily activities are not restricted. Total implantable venous access ports (TIVAPs) are increasingly used in patients undergoing long-term chemotherapy to prevent complications and problems originating from the peripheral venous system [2,3]. They are also used for other intravenous drugs, fluid replacement, and total parenteral nutrition. Access to the reservoir is achieved by passing a specially designed Huber needle through the port septum. Requiring needle insertion with each use can lead to patient discomfort and extravasation risk. In addition, these devices are relatively expensive, and insertion and removal procedures require greater technical skill; therefore, careful assessment of indication and effective patient education are important for reducing central line-associated bloodstream infections (CLABSIs) [4,5].

In TIVAP placement procedures, anesthesia and analgesia management are critical components that directly affect procedural success, in-hospital experience, and patient comfort. The standard clinical approach consists of local infiltration anesthesia with minimal sedation when necessary [6], providing a safe alternative with minimal systemic side effects while ensuring adequate analgesia during the procedure. However, more comprehensive pain control may be required in selected patient groups, and regional anesthesia techniques become important at this point [7].

Pectoral nerve blocks (PECS blocks) are ultrasound-guided interfascial plane blocks that provide analgesia to the anterior thoracic wall. Blanco et al. described blockade of the lateral and medial pectoral nerves by injecting local anesthetic into the interfascial plane between the pectoralis major and minor muscles [7]. PECS I targets the lateral and medial pectoral nerves in the plane between the pectoralis major and minor muscles, whereas PECS II adds an injection between the pectoralis minor and serratus anterior muscles to provide wider coverage, including the lateral branches of the intercostal nerves [8].

The choice of analgesia method in TIVAP placement procedures should be individualized according to patient profile, comorbidity status, operator experience, and institutional protocols. Local infiltration anesthesia represents the standard approach as a minimally invasive and safe option, providing adequate analgesia in most cases. PECS blocks offer a valuable alternative, especially in cases requiring additional support in pain management, high pain perception, or extensive dissection. The historical development of the technique and its strong anatomical foundations have ensured its significant place in regional anesthesia practice; however, more randomized controlled trials specific to TIVAP applications are needed.

A previous retrospective comparative study by Ince et al. demonstrated that ultrasound-guided PECS II block was associated with improved analgesia during central venous port catheter implantation [9]. However, several aspects of its effectiveness in routine clinical practice remain incompletely characterized, particularly procedural sedation requirements, hemodynamic responses, patient satisfaction, and whether the analgesic effect is consistent across different catheter insertion sites. Therefore, the present study sought to extend the available evidence by evaluating postoperative pain at multiple time points, together with sedation and rescue analgesic requirements, hemodynamic parameters, patient satisfaction, catheter insertion-site differences, and procedure-related complications in a retrospective clinical cohort.

## 2. Materials and Methods

### 2.1. Study Design

We conducted a retrospective observational study in accordance with the principles of the Declaration of Helsinki and reported the study in line with the STROBE recommendations. The study was approved by the Non-Interventional Research Ethics Committee of Elazığ Fethi Sekin City Hospital (decision no. 2025/12-26; 26 June 2025). Because the study used retrospective clinical files and hospital electronic records, informed consent was waived by the ethics committee. Clinical trial registration was not applicable.

### 2.2. Population

The study included patients aged 18–75 years with American Society of Anesthesiologists (ASA) physical status II or III who underwent TIVAP placement and had complete postoperative pain and analgesic records at the Anesthesiology and Reanimation Clinic of Elazığ Fethi Sekin City Hospital between 1 January 2021 and 31 December 2024. ASA physical status was extracted from the original preoperative anesthesia records, as documented during routine preanesthetic assessment, and was not retrospectively reassigned for the present study. Patients with advanced organ failure, platelet dysfunction or coagulopathy, altered consciousness or confusion, incomplete or insufficient medical records, or ASA physical status IV or V were excluded.

Patients were divided into two groups according to the anesthesia method used during TIVAP placement: Group 1 comprised patients who received local infiltration anesthesia, and Group 2 comprised patients who received ultrasound-guided PECS II block.

Only patients with complete anesthesia and postoperative pain records were included. The anesthetic technique used during port placement, including local anesthetic administration and ultrasound-guided PECS block when performed, together with postoperative Visual Analog Scale (VAS) scores, was routinely documented in clinical practice and retrospectively retrieved from the anesthesia charts and hospital electronic records.

### 2.3. Anesthesia Method

Patients underwent preanesthetic evaluation and standard monitoring in the operating room after an appropriate fasting period. The procedure area was disinfected with povidone-iodine.

During the study period, local infiltration anesthesia and ultrasound-guided PECS II block were routinely used for analgesia during port catheter placement in our department. The anesthetic technique, administered medications, and relevant procedural details were routinely documented in the anesthesia records as part of clinical practice. For the present retrospective study, these data were retrieved from the anesthesia charts and hospital electronic records.

For patients receiving local infiltration anesthesia, 30 mL of local anesthetic solution, consisting of 15 mL of 0.5% bupivacaine, 5 mL of 2% lidocaine (100 mg), and 10 mL of 0.9% saline, was infiltrated into the skin and subcutaneous tissues at the venous puncture site and port-pocket area with the patient in the supine position. If analgesia was considered inadequate, midazolam (0.03–0.1 mg/kg) and/or fentanyl (0.5–3.0 µg/kg) were administered as required.

Patients in the PECS II group received two ultrasound-guided interfascial injections. A high-frequency linear probe was positioned in the infraclavicular area to identify the pectoralis major and minor muscles and the thoracoacromial artery. According to the anesthesia records, 10 mL of the local anesthetic mixture was injected into the fascial plane between the pectoralis major and pectoralis minor muscles at the level of the third rib using an in-plane technique. The needle was then advanced, or a separate puncture was made, to the plane between the pectoralis minor and serratus anterior muscles at approximately the level of the fourth rib, where 20 mL of the same mixture was administered. The total 30 mL local anesthetic solution consisted of 15 mL of 0.5% bupivacaine, 5 mL of 2% lidocaine (100 mg), and 10 mL of 0.9% saline. During the procedure, midazolam (0.03–0.1 mg/kg) and/or fentanyl (0.5–3.0 µg/kg) was administered when analgesia or patient tolerance was considered inadequate based on the attending anesthesiologist’s clinical assessment. In the postoperative period, tramadol 50 mg was used as rescue analgesia when additional analgesic treatment was required.

After allowing sufficient time for the local anesthetic effect to develop, port catheter placement was performed. Posteroanterior chest radiography was obtained after the procedure to confirm catheter position and to assess for procedure-related complications, particularly pneumothorax.

### 2.4. Measurements

Patients’ demographic characteristics, need for additional local anesthetic, need for sedation, need for rescue analgesia, patient satisfaction, catheter depth, catheter–skin distance, and procedure-related complications were recorded. Patient satisfaction was retrieved from routine postoperative clinical records and documented as a binary outcome (satisfied/not satisfied); because the exact timing of this assessment was not consistently recorded, a specific postoperative time point could not be assigned. VAS scores were recorded at anesthesia 60 min and at postoperative 0, 1, 2, 6, 12, and 24 h. MAP was recorded at anesthesia start, anesthesia 60 min, and postoperative 2, 6, 12, and 24 h.

### 2.5. Statistical Analysis

A minimum of 102 patients in total, with at least 51 patients in each group, was determined to be required to detect a moderate effect size (Cohen’s d = 0.5) with alpha = 0.05 and power = 80%. This effect size was selected a priori as a conservative estimate because no institution-specific pilot data were available at the design stage. Using effect sizes calculated directly from our dataset, post hoc achieved power exceeded 99% for the large between-group differences observed from 60 min through 12 h (Cohen’s d = 0.90–2.86). Data were analyzed using SPSS version 23.0 (IBM Corp., Armonk, NY, USA). The normality of continuous variables was assessed using the Shapiro–Wilk test. Student’s t-test was used for normally distributed continuous variables, and the Mann–Whitney U test for non-normally distributed variables. Categorical variables were compared using the chi-square test or an exact test, as appropriate. Because of sparse cells in the multi-category primary-diagnosis table, the Fisher–Freeman–Halton exact test with Monte Carlo approximation was used for that comparison. Non-normally distributed continuous variables, including MAP and VAS, are reported as median (IQR). Potential confounders were first assessed for baseline imbalance between anesthesia groups. Given the retrospective, non-randomized design, clinically plausible covariates were entered simultaneously into the multivariable models irrespective of their univariable *p*-values. Multivariable logistic regression was performed for binary outcomes, adjusting for age, sex, ASA physical status, catheter insertion site, application side, catheter depth, and catheter–skin distance. Firth’s penalized-likelihood logistic regression was applied where complete separation occurred. VAS at postoperative 0 h was analyzed using median (quantile) regression with the same covariate set. Because VAS scores were repeatedly measured within the same patients, a generalized estimating equations (GEE) model with an exchangeable correlation structure and a group × time interaction term was additionally applied to account for within-patient correlation and evaluate whether the analgesic effect varied over time (Supplementary Table S1). A two-sided *p* < 0.05 was considered statistically significant.

## 3. Results

The final study cohort comprised 118 patients, of whom 64 (54.2%) received local infiltration anesthesia and 54 (45.8%) received ultrasound-guided PECS II block. Among the 118 patients, 67 (56.8%) were male and 51 (43.2%) were female. According to the original preoperative anesthesia records, 53 patients (44.9%) were classified as ASA physical status II and 65 (55.1%) as ASA physical status III. All port catheters were inserted for chemotherapy purposes. Colon and breast cancer were the most common primary diagnoses; rectal cancer was present in 14 patients (11.9%). The overall primary-diagnosis distribution did not differ significantly between groups (Fisher–Freeman–Halton exact test with Monte Carlo approximation, *p* = 0.280). Baseline demographic and clinical characteristics are presented in Table 1.

In the univariable assessment of potential confounders, catheter insertion site differed significantly between groups (subclavian: 54/64 [84.4%] in the infiltration group vs. 33/54 [61.1%] in the PECS II group; jugular: 10/64 [15.6%] vs. 21/54 [38.9%], respectively; *p* = 0.004). Age, sex, ASA physical status, application side, catheter depth, and catheter–skin distance were not significantly associated with anesthesia group. Catheter depth and catheter–skin distance were also similar between groups (Mann–Whitney U *p* = 0.727 and *p* = 0.105, respectively). After simultaneous adjustment for age, sex, ASA physical status, catheter insertion site, application side, catheter depth, and catheter–skin distance, PECS II block remained associated with lower odds of sedation requirement (adjusted OR 0.042, 95% CI 0.011–0.169, *p* < 0.001), additional local anesthetic use (adjusted OR 0.037, 95% CI 0.009–0.152, *p* < 0.001), and rescue analgesic requirement (adjusted OR 0.007, 95% CI 0.001–0.046, *p* < 0.001). Patient satisfaction remained associated with PECS II block in the Firth-adjusted model (adjusted OR 20.26, 95% CI 1.93–212.57, *p* = 0.012). Median quantile regression showed a 4.03-point lower adjusted median VAS at postoperative 0 h in the PECS II group (95% CI −4.64 to −3.42, *p* < 0.001). The adjusted GEE analysis confirmed a statistically significant group × time interaction for postoperative VAS (*p* < 0.001), indicating that the magnitude of the analgesic difference changed over the postoperative period (Table 2 and Table 3, Figure 1 and Figure 2).

## 4. Discussion

The results of this retrospective study suggest that PECS II block during TIVAP placement was associated with better analgesic control and greater patient comfort than local infiltration anesthesia, particularly during the early postoperative period. PECS II block was also associated with lower pain scores during the first 12 postoperative hours and with reduced requirements for sedation and rescue analgesia. The study population consisted of oncology patients undergoing TIVAP placement for chemotherapy, providing a clinically focused cohort for comparison of the two analgesic approaches.

Implantable port catheters are widely used to provide reliable venous access in applications such as long-term chemotherapy; the success rate of these interventions is high in large series, but the complication profile cannot be ignored [2,3,6]. Therefore, regional analgesia methods that both improve patient comfort and reduce systemic drug burden during port implantation are becoming increasingly important.

Although port catheter implantation is considered a relatively minor procedure, clinically relevant nociceptive stimulation may occur during skin and subcutaneous dissection, venous puncture, and subcutaneous pocket creation. In our study, VAS scores were lower in the PECS II group during the early postoperative period. The difference persisted through the first 12 postoperative hours; however, the GEE model incorporating a group × time interaction showed that the analgesic advantage progressively narrowed over time and was no longer significant by 24 h. These findings suggest that the clinical benefit of PECS II block is most pronounced during the first 12 postoperative hours. The observed analgesic pattern is consistent with previous studies and meta-analyses showing that PECS blocks reduce early postoperative pain and opioid requirements in breast surgery [10,11,12,13,14], as well as with reports of analgesic benefit during vascular access port implantation [9,15].

Sedation requirement was lower in the PECS II group than in the local infiltration group. In oncology patients with advanced age and comorbidities, sedative and opioid medications may contribute to respiratory depression, hemodynamic instability, and prolonged recovery. The lower use of additional sedative medication observed in the PECS II group may therefore be clinically relevant, although the retrospective design does not permit causal inference. Previous studies have reported reduced analgesic and sedative requirements with PECS II block in breast surgery, cardiac implantable electronic device procedures, and central venous port catheter implantation [9,15,16,17,18]. Our findings are consistent with this literature and suggest that PECS II block may be a useful analgesic option during port implantation when patient comfort and reduction in systemic medication exposure are important considerations.

The requirement for rescue analgesia was substantially lower in the PECS II group than in the infiltration group. Reducing opioid exposure may be particularly relevant in oncology patients who may already experience nausea, vomiting, appetite loss, and constipation during chemotherapy. Recent randomized studies and meta-analyses have shown that PECS blocks reduce postoperative pain and opioid requirements in breast and other anterior thoracic procedures [10,11,13,14], while studies specifically evaluating vascular access port implantation have reported similar analgesic benefits [9,15]. Importantly, the present study should be viewed as complementary rather than confirmatory evidence. The study by Ince et al. was also retrospective and demonstrated an analgesic benefit of PECS II block during port implantation [9]. Our study extends these observations by additionally evaluating sedation requirement, rescue analgesic use, patient satisfaction, hemodynamic changes, catheter insertion-site differences, and repeated postoperative pain measurements, with multivariable and repeated-measures analyses used to address potential confounding and time-dependent effects. Although PECS blocks have been shown to reduce acute postoperative pain and opioid requirements [13,14], their role in preventing persistent postsurgical pain remains uncertain [19]. We did not evaluate long-term or chronic pain outcomes; therefore, any potential longer-term benefit requires confirmation in prospective studies with extended follow-up.

MAP values were higher in the PECS II group at the recorded time points; however, no clinically relevant hemodynamic instability was documented during the study period. Because this was a retrospective comparison and hemodynamic outcomes were not the primary endpoint, these findings should be interpreted descriptively rather than as evidence of hemodynamic superiority.

Any recorded procedure-related complication occurred in 13/64 patients (20.3%) in the infiltration group and 4/54 patients (7.4%) in the PECS II group (Pearson chi-square *p* = 0.047). Individual complication types were infrequent and are therefore presented descriptively rather than as separate inferential comparisons. Given the retrospective design and sparse event frequencies, this finding should be interpreted cautiously and should not be taken as evidence that PECS II block reduces procedural complication risk. Major complications specifically attributable to PECS blocks have been reported as uncommon in previous breast surgery and CIED series [12,15,16,17,18].

Patient dissatisfaction was concentrated in the local infiltration group, while satisfaction was higher in the PECS II group. This finding was accompanied by lower early postoperative VAS scores and lower requirements for sedation and rescue analgesia. Although these associations cannot establish causality in a retrospective study, they suggest a potential patient-centered benefit of PECS II block during TIVAP placement. Previous studies have likewise reported improved patient comfort or satisfaction with PECS II block in anterior thoracic wall procedures [15,17,18].

## 5. Limitations

To address the non-randomized nature of the study, multivariable-adjusted analyses were performed for sedation requirement, additional local anesthetic use, rescue analgesic need, patient satisfaction, and VAS score at postoperative 0 h. The models were adjusted for age, sex, ASA physical status, catheter insertion site, application side, catheter depth, and catheter–skin distance. Catheter insertion site was the only candidate variable showing a statistically significant baseline imbalance between groups; nevertheless, the associations between PECS II block and the principal clinical outcomes remained statistically significant after simultaneous adjustment for all clinically plausible covariates (Table 4). Residual confounding and indication bias cannot be completely excluded because of the retrospective, non-randomized design. In addition, sedation and rescue medication were administered according to the attending anesthesiologist’s clinical assessment, patient satisfaction was recorded as a binary outcome without a standardized assessment time, and the low number of individual complications limits inference regarding safety outcomes.

## 6. Conclusions

In this retrospective cohort, PECS II block was associated with lower postoperative pain scores during the early postoperative period and reduced requirements for sedation and rescue analgesia compared with local infiltration anesthesia. These findings suggest that PECS II block may be a useful component of multimodal analgesia for TIVAP placement. Prospective randomized studies are needed to confirm these observations and to better define safety, patient-centered outcomes, and the duration of analgesic benefit.

## Figures and Tables

**Figure 1:**
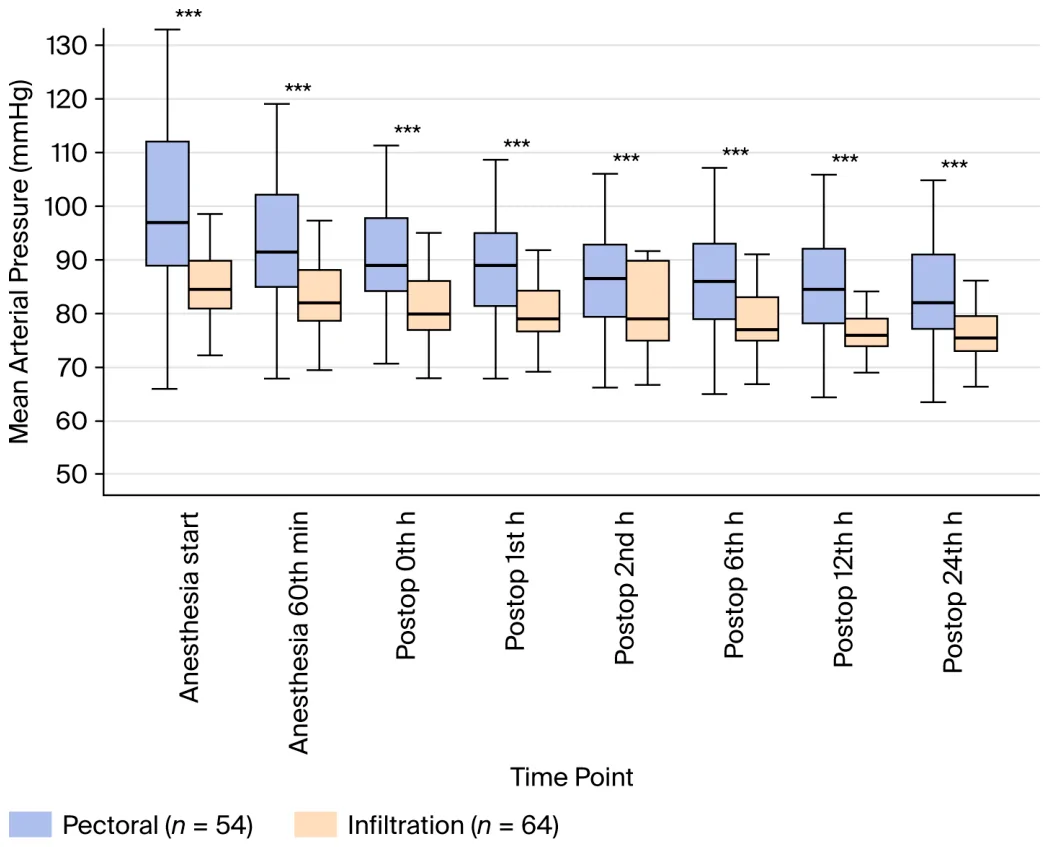
Mean arterial pressure analysis: MAP in the PECS II block (*n* = 54) versus local infiltration (*n* = 64) groups. Box plots display median, IQR, and whiskers. Unadjusted Mann–Whitney U comparisons showed higher MAP values in the PECS II group at the recorded time points (*** *p* < 0.001); these findings are interpreted descriptively.

**Figure 2:**
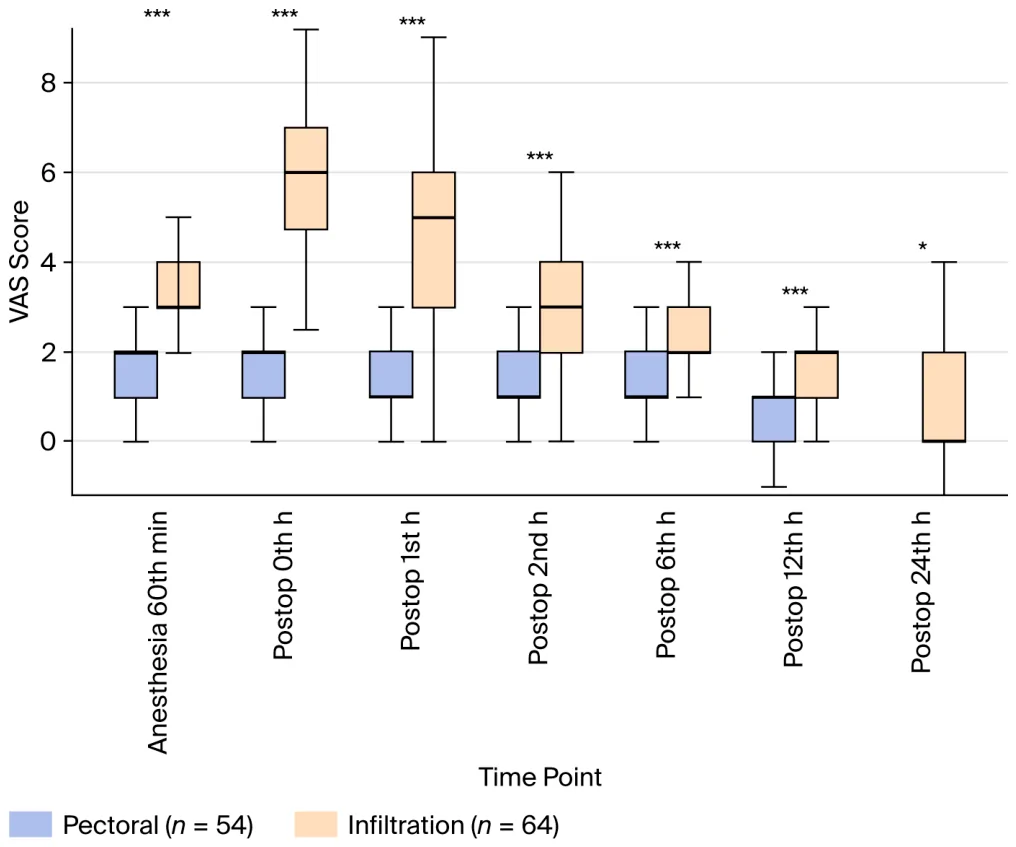
Visual Analog Scale (VAS) analysis: postoperative pain scores in PECS II block (*n* = 54) versus local infiltration (*n* = 64) groups. Box plots show median, IQR, and whiskers (1.5 × IQR). Unadjusted Mann–Whitney U comparisons favored the PECS II group across recorded time points; the repeated-measures GEE analysis showed that the adjusted analgesic advantage diminished over time and was no longer significant by 24 h (Supplementary Table S1). Statistical significance is indicated as follows: * *p* < 0.05 and *** *p* < 0.001.

**Table 1:** Baseline demographic and clinical characteristics of the study population.

Variables	Infiltration (*n* = 64)	PECS II Block (*n* = 54)	*p*-Value
Age (years), Median (IQR)	61.0 (21.0)	61.5 (12.0)	0.497
Gender, *n* (%)			0.617
Male	35 (54.7%)	32 (59.3%)	
Female	29 (45.3%)	22 (40.7%)	
ASA Status, *n* (%)			0.227
II	32 (50.0%)	21 (38.9%)	
III	32 (50.0%)	33 (61.1%)	
Primary Diagnosis, *n* (%)			0.280
Colon Cancer	13 (20.3%)	22 (40.7%)	
Breast Cancer	14 (21.9%)	6 (11.1%)	
Gastric Cancer	8 (12.5%)	6 (11.1%)	
Lung Cancer	7 (10.9%)	6 (11.1%)	
Sarcoma	4 (6.3%)	1 (1.9%)	
Pancreatic Cancer	2 (3.1%)	3 (5.6%)	
Esophageal Cancer	2 (3.1%)	0 (0.0%)	
Mesothelioma	2 (3.1%)	0 (0.0%)	
Rectal Cancer	8 (12.5%)	6 (11.1%)	
Others *	4 (6.3%)	4 (7.4%)	

Abbreviations: ASA, American Society of Anesthesiologists; IQR, interquartile range. Note: Data are presented as *n* (%) or median (IQR). * Others includes Brain Tm, Hepatic Ca, Laryngeal Ca, Lymphoma, Nasopharyngeal Ca, Ovarian Ca, and Prostate Ca. The primary-diagnosis distribution was compared using the Fisher–Freeman–Halton exact test with Monte Carlo approximation because of sparse cells (*p* = 0.280). Other *p*-values represent comparisons between the infiltration and PECS II block groups.

**Table 2:** Procedural and operative characteristics.

Variables	Infiltration (*n* = 64)	PEC II Block (*n* = 54)	*p*-Value
Application Side, *n* (%)			0.976
Right	57 (89.1%)	48 (88.9%)	
Left	7 (10.9%)	6 (11.1%)	
Catheter Site, *n* (%)			**0.004**
Subclavian	54 (84.3%)	33 (61.1%)	
Jugular	10 (15.6%)	21 (38.9%)	
Additional Local Anesthesia, *n* (%)			**<0.001**
Required (Yes)	49 (76.6%)	22 (40.7%)	
Not Required (No)	15 (23.4%)	32 (59.3%)	
Sedation Requirement, *n* (%)			**<0.001**
Required (Yes)	36 (56.3%)	6 (11.1%)	
Not Required (No)	28 (43.7%)	48 (88.9%)	
Catheter Depth (cm), Median (IQR)	14.0 (1.0)	14.0 (3.0)	0.727
Catheter–Skin Distance (cm), Median (IQR)	20.0 (2.0)	21.0 (2.0)	0.105

Abbreviations: IQR, interquartile range; *n*, number of patients. Categorical variables are presented as frequencies and percentages [*n* (%)]. Continuous variables (Catheter Depth and Catheter–Skin Distance) are presented as median (IQR) due to their non-normal distribution. *p*-values were calculated using the Pearson Chi-square test for categorical variables and the Mann–Whitney U test for continuous variables; bold *p*-values indicate statistical significance at the *p* < 0.05 level. The source-verified Mann–Whitney U *p*-values were 0.727 for catheter depth and 0.105 for catheter–skin distance.

**Table 3:** Clinical outcomes: Pain scores, hemodynamics, and complications.

Variables	Infiltration (*n* = 64)	PECS II Block (*n* = 54)	*p*-Value
Pain Scores (VAS), Median (IQR)			
VAS 60 min	3.0 (1.0)	2.0 (1.0)	**<0.001**
Postop 0 h	6.0 (3.0)	2.0 (1.0)	**<0.001**
Postop 1 h	5.0 (3.0)	1.0 (1.0)	**<0.001**
Postop 2 h	3.0 (2.0)	1.0 (1.0)	**<0.001**
Postop 6 h	2.0 (1.0)	1.0 (1.0)	**<0.001**
Postop 12 h	2.0 (1.0)	1.0 (1.0)	**<0.001**
Postop 24 h	0.0 (2.0)	0.0 (0.0)	**0.037**
Hemodynamics (MAP, mmHg), Median (IQR)			
MAP 0 min	84.5 (10.0)	97.0 (24.0)	**<0.001**
MAP 60 min	82.0 (10.0)	91.5 (18.0)	**<0.001**
Postop 2 h	79.0 (9.0)	86.5 (14.0)	**<0.001**
Postop 6 h	77.0 (8.0)	86.0 (14.0)	**<0.001**
Postop 12 h	76.0 (5.0)	84.5 (14.0)	**<0.001**
Postop 24 h	75.5 (8.0)	82.0 (14.0)	**<0.001**
Patient Satisfaction, *n* (%)			**<0.001**
Satisfied	52 (81.3%)	54 (100%)	
Not Satisfied	12 (18.8%)	0 (0%)	
Additional Analgesic Need, *n* (%)			**<0.001**
Yes	53 (82.8%)	6 (11.1%)	
No	11 (17.2%)	48 (88.9%)	
Any complication, *n* (%)			0.047
Yes	13 (20.3%)	4 (7.4%)	
No	51 (79.7%)	50 (92.6%)	
Complication types (descriptive)			
Hematoma/infection/pneumothorax	4/1/2	1/1/0	
Other *	6 (9.4%)	2 (3.7%)	

Abbreviations: IQR, interquartile range; *n*, number of patients. Categorical outcomes are presented as frequencies and percentages [*n* (%)]. VAS scores are reported at anesthesia 60 min and postoperative 0, 1, 2, 6, 12, and 24 h; MAP is reported at anesthesia start, anesthesia 60 min, and postoperative 2, 6, 12, and 24 h. Continuous outcomes are presented as median (IQR). Mann–Whitney U tests were used for the continuous comparisons. The *p* value for ‘Any complication’ compares the presence versus absence of any recorded complication using the Pearson chi-square test (13/64 vs. 4/54; *p* = 0.047). Individual complication types are shown descriptively because event counts were small. * Other includes catheter blockage, catheter change/revision, malposition, and bleeding as documented in the source data. Bold values indicate statistical significance (*p* < 0.05).

**Table 4:** Adjusted Analysis: Multivariable-Adjusted Associations Between PECS II Block and Clinical Outcomes (PECS II block vs. local infiltration, reference).

Outcome	Crude OR (95% CI)	Adjusted OR (95% CI) *	*p* (adj.)	Method
Sedation requirement	0.105 (0.040–0.274)	0.042 (0.011–0.169)	<0.001	Logistic regression
Additional local anesthesia	0.217 (0.098–0.478)	0.037 (0.009–0.152)	<0.001	Logistic regression
Additional analgesic need	0.029 (0.010–0.082)	0.007 (0.001–0.046)	<0.001	Logistic regression
Patient satisfaction	25.95 (1.46–461.5)	20.26 (1.93–212.57)	0.012	Firth penalized logistic †

* Adjusted for age, sex, ASA physical status (III vs. II), catheter insertion site (subclavian vs. jugular), application side, catheter depth, and catheter–skin distance using multivariable logistic regression. † Firth’s penalized-likelihood logistic regression was used for patient satisfaction because of complete separation (0/54 dissatisfied cases in the PECS II group). A median (quantile) regression for VAS at postoperative 0 h, adjusted for the same covariates, showed a 4.03-point lower median VAS in the PECS II group (95% CI −4.64 to −3.42, *p* < 0.001).

## Data Availability

The original contributions presented in this study are included in the article/Supplementary Materials. Further inquiries can be directed to the corresponding author.

## Supplementary Materials

The following supporting information can be downloaded at https://www.mdpi.com/article/10.3390/medicina62101877/s1, Table S1: Postoperative VAS scores over time, with repeated-measures (GEE) adjustment.
