# Supplementary material for: Analgesic Effectiveness of Ultrasound-Guided PECS II Block in Implantable Venous Port Catheter Placement: A Retrospective Comparative Study
Source: Medicina (Kaunas). 2026 Sep 28;62(10):1877. doi: 10.3390/medicina62101877 (PMC13706861; doi:10.3390/medicina62101877)
Supplement: Supplementary file 1 [file medicina-62-01877-s001.zip › medicina-4534033-supplementary.pdf]

Supplementary Table S1. Postoperative VAS scores over time, with repeated-measures (GEE) adjustment.

Variables	Infiltration (n = 64)	Pectoral Block (n = 54)	p-Value	Holm-adjusted p	GEE net group difference*
**Postop 0 h**	6.0 (3.0)	2.0 (1.0)	**<0.001**	**<0.001**	−2.70
**Postop 1 h**	5.0 (3.0)	1.0 (1.0)	**<0.001**	**<0.001**	−2.59
**Postop 2 h**	3.0 (2.0)	1.0 (1.0)	**<0.001**	**<0.001**	−2.47
**Postop 6 h**	2.0 (1.0)	1.0 (1.0)	**<0.001**	**<0.001**	−2.00
**Postop 12 h**	2.0 (1.0)	1.0 (1.0)	**<0.001**	**<0.001**	−1.29
**Postop 24 h**	0.0 (2.0)	0.0 (0.0)	**0.037**	**0.037**	+0.13

Abbreviations: IQR, interquartile range; GEE, generalized estimating equations. Data are presented as median (IQR), consistent with Table 3. Unadjusted p-values were calculated using the Mann–Whitney U test; Holm correction was applied for multiple comparisons across the six time points; bold p-values indicate statistical significance at the $p < 0.05$ level. **GEE net group difference = Pectoral Block − Infiltration, derived from a generalized estimating equations model (exchangeable correlation structure) including a group×time interaction term. Negative values indicate lower (better) VAS scores in the Pectoral Block group.*

Note: The group×time interaction was statistically significant ($\beta = 0.118$; $p<0.001$), indicating that the analgesic advantage of the PECS block, while pronounced in the early postoperative period, diminished progressively over time and was no longer significant by 24 hours.
